# Experience with a novel high frequency optical coherence tomography device for intracoronary imaging: a case series

**DOI:** 10.3389/fcvm.2024.1384222

**Published:** 2024-06-07

**Authors:** Dorian Garin, Stéphane Cook, Mario Togni, Pascal Meier, Peter Wenaweser, Serban Puricel, Diego Arroyo

**Affiliations:** Department of Cardiology, University and Hospital Fribourg, Fribourg, Switzerland

**Keywords:** optical coherence tomography, percutaneous coronary intervention, intravascular imaging, coronary artery disease, image quality assessment

## Abstract

**Introduction:**

Intravascular imaging, especially optical coherence tomography (OCT), has significantly improved percutaneous coronary intervention (PCI), yet its routine clinical application faces challenges. This case series introduces the Gentuity® High-Frequency Optical Coherence Tomography (HF-OCT), a novel device designed to enhance intracoronary imaging with a significantly faster pullback and smaller catheter size, potentially offering enhanced navigability in complex lesions. We aimed to assess the image quality of Gentuity® HF-OCT in complex vessel conditions, as well as presenting a case series to illustrate the application of the device in various clinical scenarios.

**Methods:**

In this case series, we included all patients who underwent intracoronary HF-OCT imaging at our center. The primary endpoint was image quality assessed by clear image length (CIL). Image quality was assessed in relation to (1) lesion severity assessed by minimum lumen area (MLA); (2) vessel size, differentiating between larger (diameter ≥ 4 mm) and smaller vessel segments; (3) pre- vs. post-PCI conditions, and (4) vessel tortuosity, categorized into none, moderate, and severe.

**Results:**

Twenty-four HF-OCT runs from 14 patients were included. No significant differences in CIL were observed across lesion severity terciles (*p* = 0.449), between small and large vessel segments [mean CIL% difference 1.3%; confidence interval (CI), −9.3 to 11.8; *p* = 0.802], and pre- vs. post-PCI conditions (mean CIL difference −3.9 mm; CI, −14.0 to 6.1; *p* = 0.373). Vessel tortuosity significantly impacted image quality, with clear reductions in CIL observed in cases of moderate (74.8; CI, 73.5 to 76.0; vs. 63.9; CI, 56.2 to 71.5; *p* = 0.043) and severe tortuosity (74.8; CI, 73.5 to 76.0; vs. 65.0; CI, 62.1 to 67.9; *p* = 0.002) compared to vessels with no tortuosity. Overall, the HF-OCT demonstrated excellent catheter deliverability and crossability, with very satisfactory image quality and no significant adverse events.

**Conclusion:**

The Gentuity® HF-OCT is a new OCT device capable of navigating both small- and large-diameter vessels, with similar image quality, but vessel tortuosity seems to have an impact on image quality. It appears to be as usable as conventional OCT for pre-PCI diagnosis and OCT-guided PCI, potentially bringing additional benefits in terms of deliverability, lesion crossover and ease of use in routine clinical practice.

## 1 Introduction

Intravascular imaging plays a pivotal role in modern percutaneous coronary intervention (PCI), improving procedural success and long-term outcomes in patients with coronary artery disease (CAD) (1–4). Among the different intravascular imaging modalities, optical coherence tomography (OCT), which uses infrared light, provides detailed assessment of the vascular lumen, the vessel structure, plaque morphology and stent visualization (5). It has recently been shown to be non-inferior to intravascular ultrasound (IVUS)-guided PCI for clinical outcome (6) and was associated with a larger minimum stent area (MSA) compared to angiography-guided PCI (7). OCT-guided bifurcation PCI is associated with a lower incidence of major adverse cardiac events at 2 years (8). Despite guideline recommendations (9, 10), intravascular imaging has not been adopted in routine clinical practice for various reasons including: time and financial constraints, the need for proper training in image interpretation and issues on increased use of contrast (3). In this case series, we introduce a novel OCT device, the Gentuity® High-Frequency Optical Coherence Tomography (HF-OCT, Nipro Medical).

This new-generation HF-OCT device displays technical features that distinguish it from standard OCT. The imaging catheter profile of 1.8-French is twice times smaller than that of standard OCT (11). It features a pullback speed of 100 mm/s, almost three times that of conventional OCT imaging systems, which operate at a speed around 36 mm/s (12). HF-OCT images can therefore be acquired in a considerably shorter time, on the order of one second for 100 mm pullbacks. The HF-OCT lens uses a 1.310 nm wavelength infrared light with an extended scan range over larger vessels up to 7 mm, expanding its applicability to large left main coronary arteries, renal and even pulmonary arteries. Acquisition speed is 250 cross-sectional images per second with an axial resolution of 10 µm for a refractive index of 1.4. Maximum pullback length is 100 mm. The catheter is a total of 165 cm long making it an optimal length for radial access.

HF-OCT is predominantly utilized in interventional neuroradiology, where it could generate for the first-time high-quality images of small, tortuous cerebral vessels (11, 12). The first intra-human study for coronary imaging has recently been published, showing the ability to image long, severely stenotic coronary segments without any preparation, and a preservation of image quality even in stenotic lesions (13). A comparison between HF-OCT and conventional OCT in identical vessels indicated that HF-OCT provides similar imaging quality but requires less contrast (14). The clinical benefits of the HF-OCT catheter lie in its enhanced ability to cross extremely narrow lesions inaccessible to standard OCT catheters, its greater flexibility in navigating through tortuous arterial pathways, and its capacity to scan a broader spectrum of vessel diameters while offering faster pullback, which could reduce the bias caused by cardiac movements (12). Since conventional OCT catheters are not generally used for imaging such challenging vascular conditions, it is not known whether the imaging quality of HF-OCT persists in complex lesions. The aim of this case series is to assess the feasibility of using this device in an everyday catheterization laboratory. We hypothesized that pullback image quality would persist regardless of whether the catheter imaged a severely stenotic lesion, a larger vessel, in pre- vs. post-PCI settings, or a tortuous vessel. To test this hypothesis, we evaluated image quality in a variety of real-life clinical scenarios and provided illustrative cases of the capabilities of HF-OCT.

## 2 Materials and methods

### 2.1 Study population

This is a retrospective, single arm, monocentric study including all consecutive patients undergoing planned or urgent PCI with intracoronary HF-OCT. Inclusion criteria were at least one HF-OCT run, and participation in the Cardio-FR registry. Decision to perform intravascular imaging using OCT was at the operator discretion.

The Cardio-FR (NCT04185285) registry at University and Hospital Fribourg includes all patients aged 18 and above who are admitted for PCI and have provided written informed consent. The registry collects comprehensive data on baseline patient characteristics, procedural details, in-hospital outcomes, and annual clinical follow-up. The Cardio-FR registry complies with the Helsinki Declaration and is approved by the local ethics committee (003-REP-CER-FR).

### 2.2 Device and intervention

**Figure 1:**
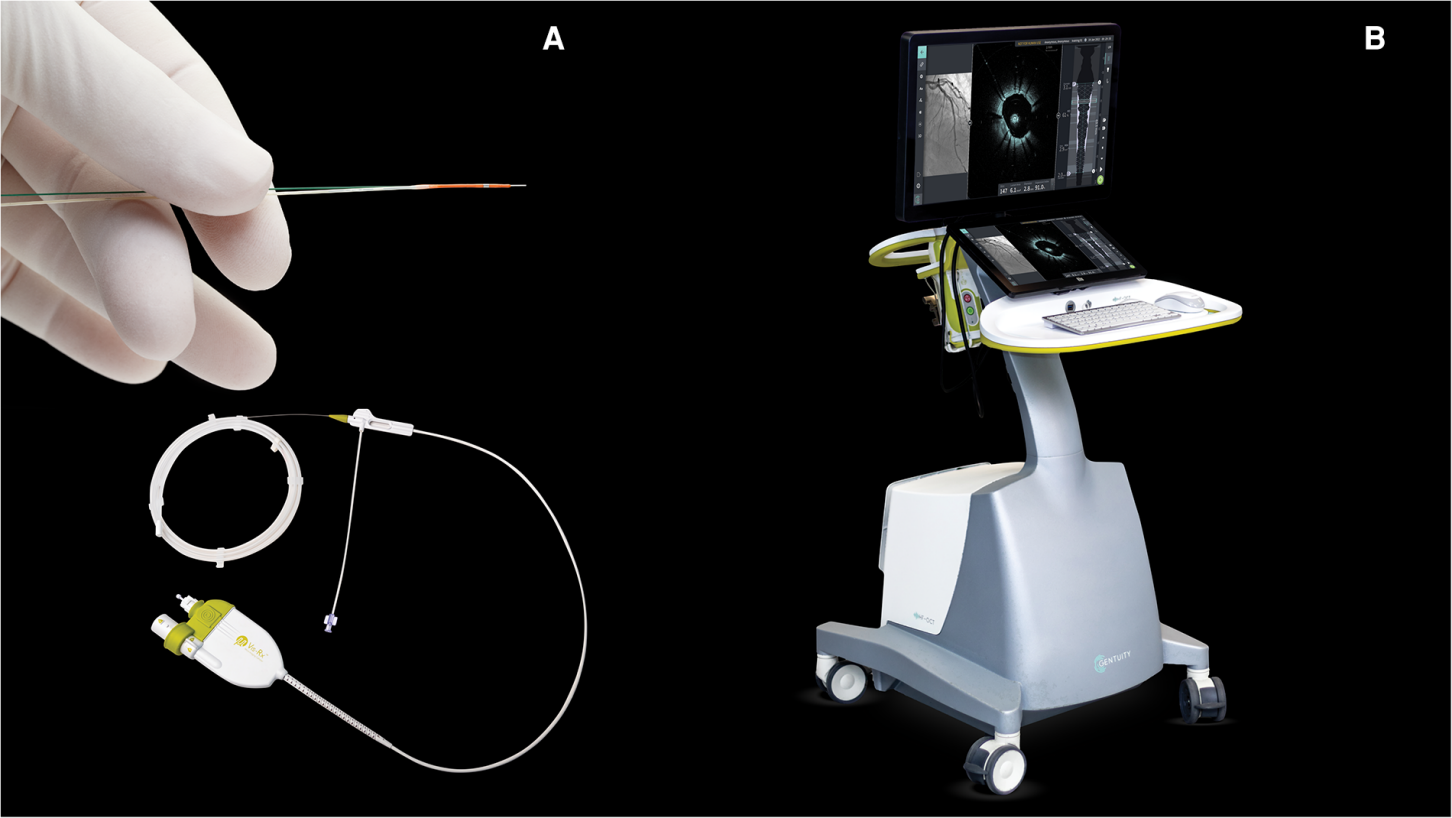
(**A**) Rapid exchange 1.8-French (0.6 mm) microcatheter (Vix-Rx Micro-Imaging Catheter, Gentuity, Nipro Medical Europe N.V.). (**B**) HF-OCT console (Gentuity, Nipro Medical Europe N.V.). © Nipro Medical Europe N.V., with permission. HF-OCT, high-frequency optical coherence tomography.

**Figure 2:**
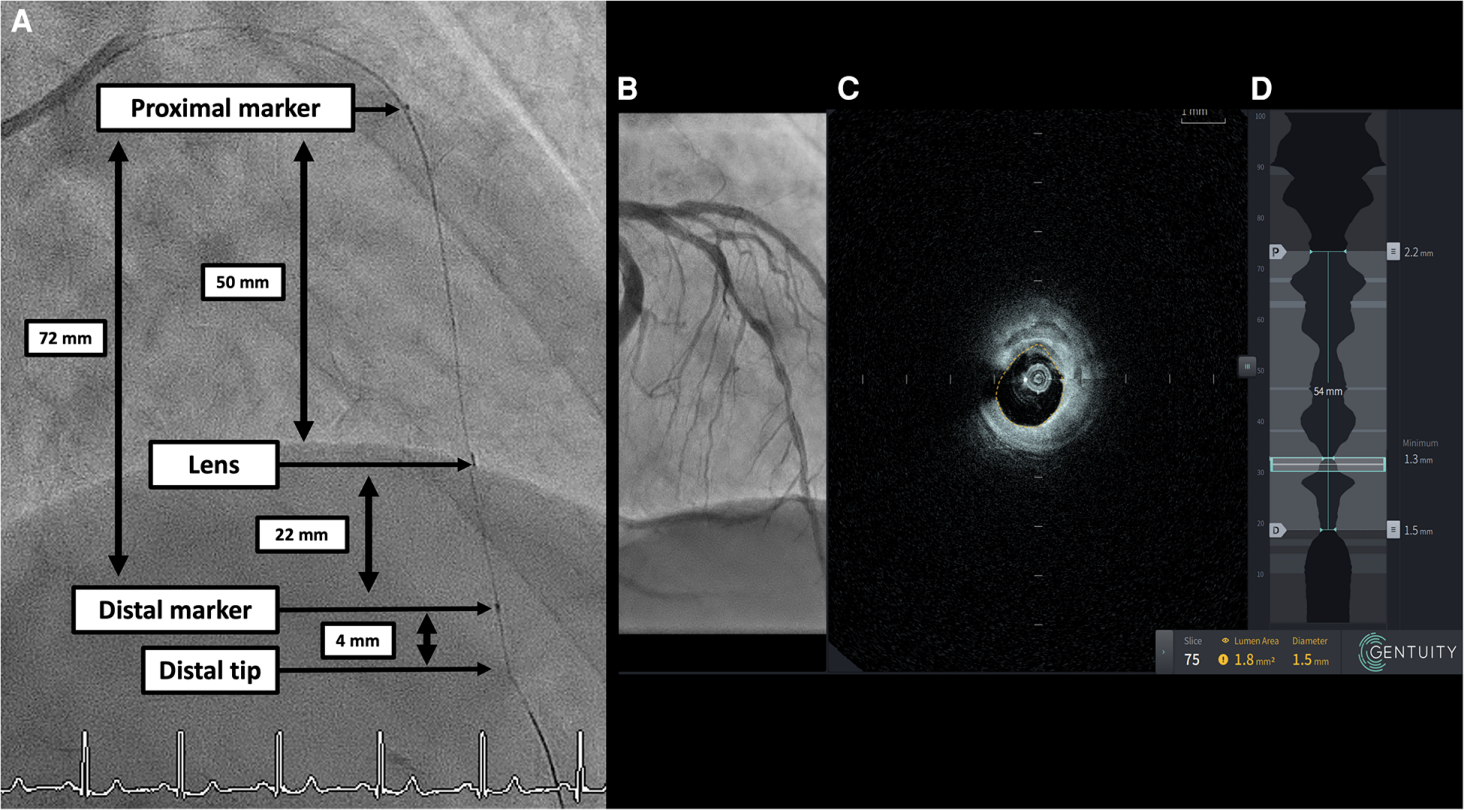
(**A**) Angiographic still frame with no contrast depicting the Gentuity HF-OCT catheter in the LAD. Distal and proximal marker are 72 mm from each other. The lens is situated at 26 mm from the catheter tip, 22 mm from the distal marker and 50 mm from the proximal marker. The total pullback length is 100 mm. (**B**) Angiography co-registration. (**C**) Corresponding OCT cross-section with automatic lumen detection. (**D**) Longitudinal view of a 100 mm pullback with automatic lumen detection, lumen area and diameter. HF-OCT, high-frequency optical coherence tomography; LAD, left anterior descending artery.

Procedures were performed via either radial or femoral access with a 6-French guiding catheter. Pre-procedural antithrombotic regimen was systematically achieved with aspirin (250–500 mg i.v. bolus for those not under treatment and 100 mg for those already under aspirin and then 100 mg/day for all) and unfractionated heparin (70 UI/kg). All patients received a minimum of 300 µg of i.v. nitroglycerine prior to intravascular imaging. The Gentuity® HF-OCT device was calibrated to the angiography for co-registration purposes. This device uses a rapid exchange 1.8-French (0.6 mm) microcatheter (Vix-Rx Micro-Imaging Catheter, Gentuity) (Figure 1A) connected to the console (Figure 1B). It was prepared as per user instructions and flushed with saline prior to insertion through the Y-connector. Proper HF-OCT catheter positioning was checked on angiography, specifically, the lens was positioned distal to the region of interest (Figure 2). The catheter was flushed prior to pullback, and a test contrast puff ensured proper lumen clearance and guide-catheter positioning. The trigger of the HF-OCT system was set on automatic contrast detection and the contrast was delivered by contrast injector ACIST CVi (5 ml/s, 12 ml, 300 psi 0.0 s). The injection was interrupted as soon as the guiding catheter was visualized on HF-OCT console. All pullbacks were at a speed of 100 mm/s. Runs were repeated as necessary, particularly in cases of guided PCI and stent optimization.

### 2.3 Study definitions

HF-OCT runs were analyzed during and after the procedure for pre-PCI plaque morphology, vessel dimensions and all relevant anatomical features. HF-OCT was used for PCI planification, strut visualisation and post stenting optimization. All runs and angiography were then reviewed by two blinded operators (DG and DA). The quality of HF-OCT images was evaluated by clear image length (CIL, mm), defined by a minimum of 270° of the vessel circumferences distinctly discernible (15). A good-quality image was considered to have a CIL% ≥ 80% (15). Minimum lumen area (MLA, mm^2^) was defined by the smallest cross-sectional area of the vessel calculated by the software.

Vessels were stratified for tortuosity, determined on the number and severity of curves navigated by the balloon or device to access the target lesion. These curves were identified in the end-diastolic frame, focusing on segments with the sharpest angle. Vessel tortuosity was classified into three categories: no tortuosity, characterized by fewer than two curves, each less than 90 degrees, required to reach the target lesion; moderate tortuosity: defined as the necessity to navigate either two curves greater than 75 degrees or a single curve exceeding 90 degrees to access the target lesion; and severe tortuosity, involving either two curves greater than 90 degrees or three curves each exceeding 75 degrees to reach the target lesion (16).

When a run included a HF-OCT calculated diameter ≥ 4 mm, it was further stratified into two categories: large vessel segment (diameter ≥ 4 mm) and small vessel segment (diameter < 4 mm). Clear image length proportion (CIL%) was calculated for each segment as CIL divided by segment length and displayed in percentage.

### 2.4 Statistical rational and analysis

The primary endpoint was the quality of the HF-OCT run assessed by CIL. Image quality was analyzed in 4 ways. (1) To determine the effect of lesion severity on image quality, pullbacks were divided into three terciles based on MLA. We then analyzed the differences in CIL among these terciles using the Kruskal-Wallis test. Post-hoc Mann-Whitney *U* tests were conducted to identify specific group differences. (2) For each vessel that was further divided into large and small segment, we computed the difference in mean CIL% using a paired sample *T*-test. (3) Mean CIL difference between pre- and post-PCI was calculated for each vessel that had HF-OCT-guided-PCI using a paired-sample *T*-test. (4) To assess the impact of vessel tortuosity on CIL, we grouped the vessels into categories of no, moderate, or severe tortuosity and used the Kruskal-Wallis and post-hoc Mann-Whitney *U* tests.

Normality and homogeneity of variances was determined visually, with the Shapiro-Wilk test and Levene's test, as appropriate. A *p*-value of less than 0.05 was considered statistically significant. Continuous variables were reported as mean ± standard deviation or as median (interquartile range) if skewed. Categorical variables were reported as count (percentages). All statistical analyses and representations were performed using R (version 4.3.2, © 2023 by *The R foundation)* and R Studio (Version 2023.09.1+494, © 2022 by *Posit Software, PBC*).

## 3 Results

Between June, 20 and July, 7 2023, 14 patients (16 vessels, 24 pullbacks) undergoing either elective or urgent PCI had an HF-OCT run and were included. None were excluded. Table 1 shows baseline characteristics of patients and vessels. A total of 5 (35.7%) patients underwent urgent PCI for acute coronary syndrome (ACS). Indications for the use of HF-OCT were 11 (68.8%) for diagnosis and HF-OCT-guided PCI, and 5 (31.3%) for diagnosis only (Supplementary Table S1). Of importance, there were no adverse events, no mechanical device failures, and no complications such as arrhythmia, embolisation, coronary dissection or spasm.

**Table 1:** Baseline patient and lesion characteristics.

Baseline patient's characteristics	
Age (years)	65.5 [57.5–73.5]
Male sex	11 (78.6)
Known CAD	13 (92.9)
Diabetes	2 (14.2)
Hypertension	5 (35.7)
Dyslipidemia	13 (92.9)
Active tobacco smoking	3 (21.4)
Procedural characteristics	
Indication	
Stable CAD NSTEMI Unstable angina	9 (64.3) 3 (21.4) 2 (14.3)
Vessels imaged LMCA LAD LCx RCA	16 (100) 2 (12.5) 12 (75.9) 0 (0.0) 2 (12.5)
Radial access	10 (71.4)
Femoral access	4 (28.6)
Mean procedure radiation time (min)	7.41 ± 3.33
Mean total amount of contrast use (ml)	134.1 ± 54.66
Baseline measurements of HF-OCT images	
Minimal luminal area (mm^2^)	2.85 [1.9–4.6]
Minimal lumen diameter (mm)	1.60 [1.6–2.45]
Median pullback length, excluding guide catheter (mm)	78.8 [72.3–85.3]

Values are *n* (%), mean ± standard deviation, or median [interquartile range]. CAD, coronary artery disease; CCS, chronic coronary syndrome; CIL, clear image length; NSTEMI, non-ST-elevation myocardial infarction; LAD, left anterior descending artery; LCx, left circumflex artery; LMCA, left main coronary artery; RCA, right coronary artery.

On average, CIL was 69.0 mm (60.3–74.1), representing 82.1% (71.8–88.2) of the HF-OCT acquisition, excluding the guide catheter. 7 (29.1%) runs did not meet the required criteria for good image quality. The smallest MLA was 0.4 mm^2^ in a 0.7 mm diameter lesion. The average amount of contrast used per run was 6.3 ± 0.8 ml.

**Figure 3:**
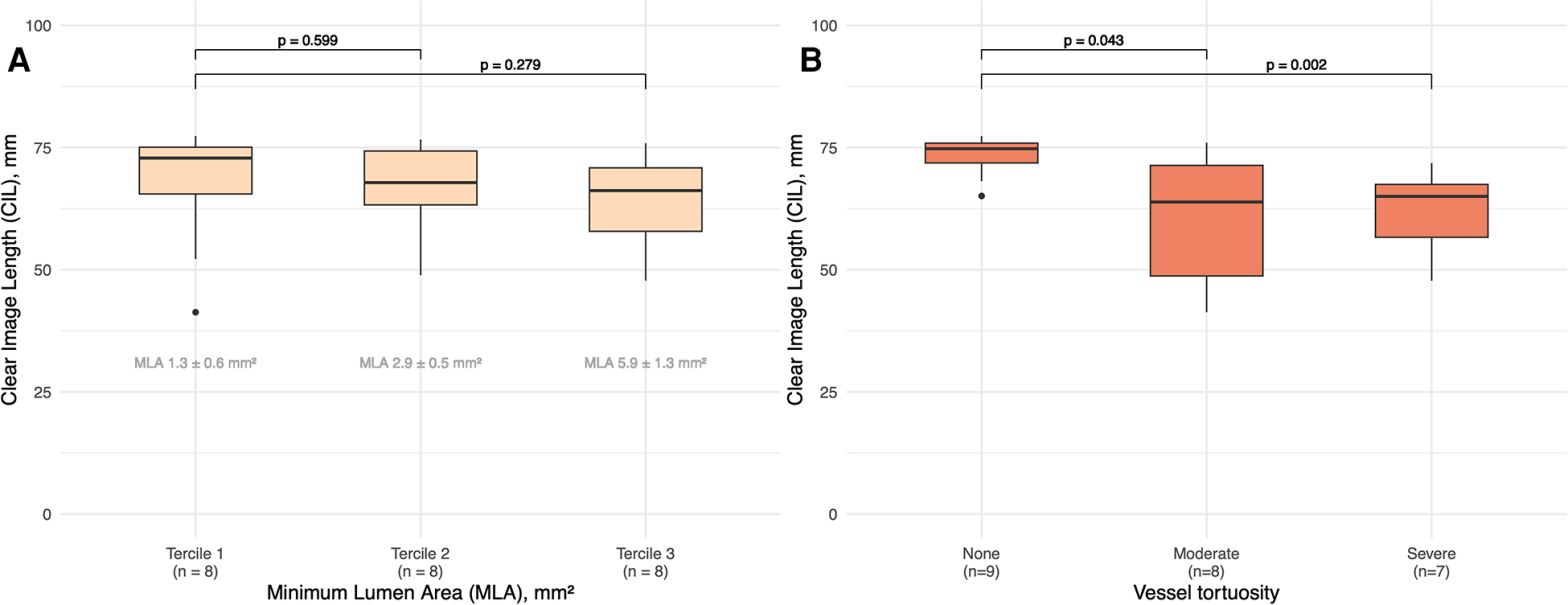
(**A**) Boxplots showing clear image length for terciles of MLA. *P* values between the terciles are shown. Mean ± standard deviation of MLA for each tercile is shown. MLA, minimum lumen aera. (**B**) Boxplots showing clear image length for tortuosity categories (none, moderate, severe). *P* values between the categories are shown.

There was no significant difference in CIL between the different MLA terciles (*p* = 0.449). Post-hoc comparison between the tercile with the lowest MLA and the higher terciles revealed no significant differences in image quality, as shown in Figure 3A.

A total of 17 (70.8%) pullbacks had a segment containing a large vessel (diameter ≥ 4 mm), with a mean length of 13.8 ± 10.1 mm. In these cases, no significant difference was found in mean CIL% between small-diameter vs. large-diameter segments [mean CIL% difference 1.3%; confidence interval (CI), −9.3 to 11.8; *p* = 0.802]. The largest lesion imaged without pre-dilatation was at the ostium of the LAD, angiographically 70%–80% stenotic, with an MLA of 3.3 mm^2^ and a CIL% of 95.6%. Comparing pre- and post-intervention CIL for the same arteries, there was no significant difference in mean CIL between pre-PCI (without predilatation) and post-PCI pullbacks (mean CIL difference −3.9 mm; CI, −14.0 to 6.1; *p* = 0.373).

Analysis of the impact of different levels of vascular tortuosity (none, moderate or severe) on image quality revealed a statistically significant variation between tortuosity categories (*p* = 0.012), confirmed by the post-hoc Mann-Whitney *U* test. A significant difference was observed in median CIL values between vessels without tortuosity and those with severe tortuosity (74.8; CI, 73.5–76.0; vs. 65.0; CI, 62.1–67.9; *p* = 0.002). A similar significant difference was observed between vessels without tortuosity and those with moderate tortuosity (74.8; CI, 73.5–76.0; vs. 63.9; CI, 56.2–71.5; *p* = 0.043). However, there was no significant difference in median CIL between the moderate and severe tortuosity groups (*p* = 0.867), as shown in Figure 3B.

**Figure 4:**
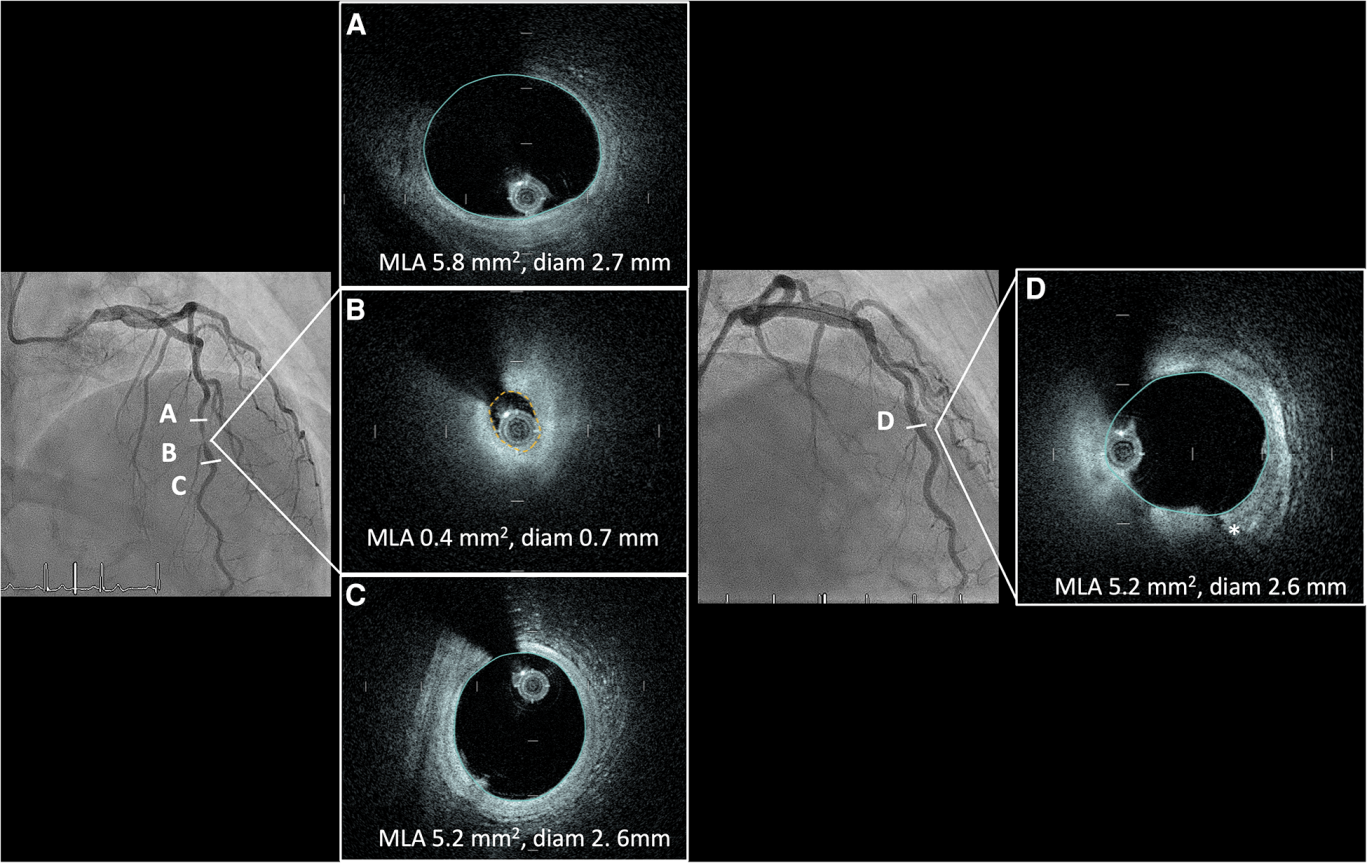
Angiographic LAD cranial view depicting a very tight stenosis of the distal LAD (**B**) with corresponding OCT cross-sections showing a fibro-lipidic plaque with a minimal lumen area of 0.4 mm2. Good proximal (**A**) and distal (**C**) visualisation of the lumen and components of the vessel wall. (**D**) Angiographic LAD cranial view in the same patient post angioplasty with Wolverine cutting balloon 2.5 mm and stepwise NC balloon escalation to 3.0 prior to final treatment with drug coated balloon. Controlled dissection plane from the cutting balloon (*). HF-OCT, high-frequency optical coherence tomography; LAD, left anterior descending artery; NC, non-compliant.

**Figure 5:**
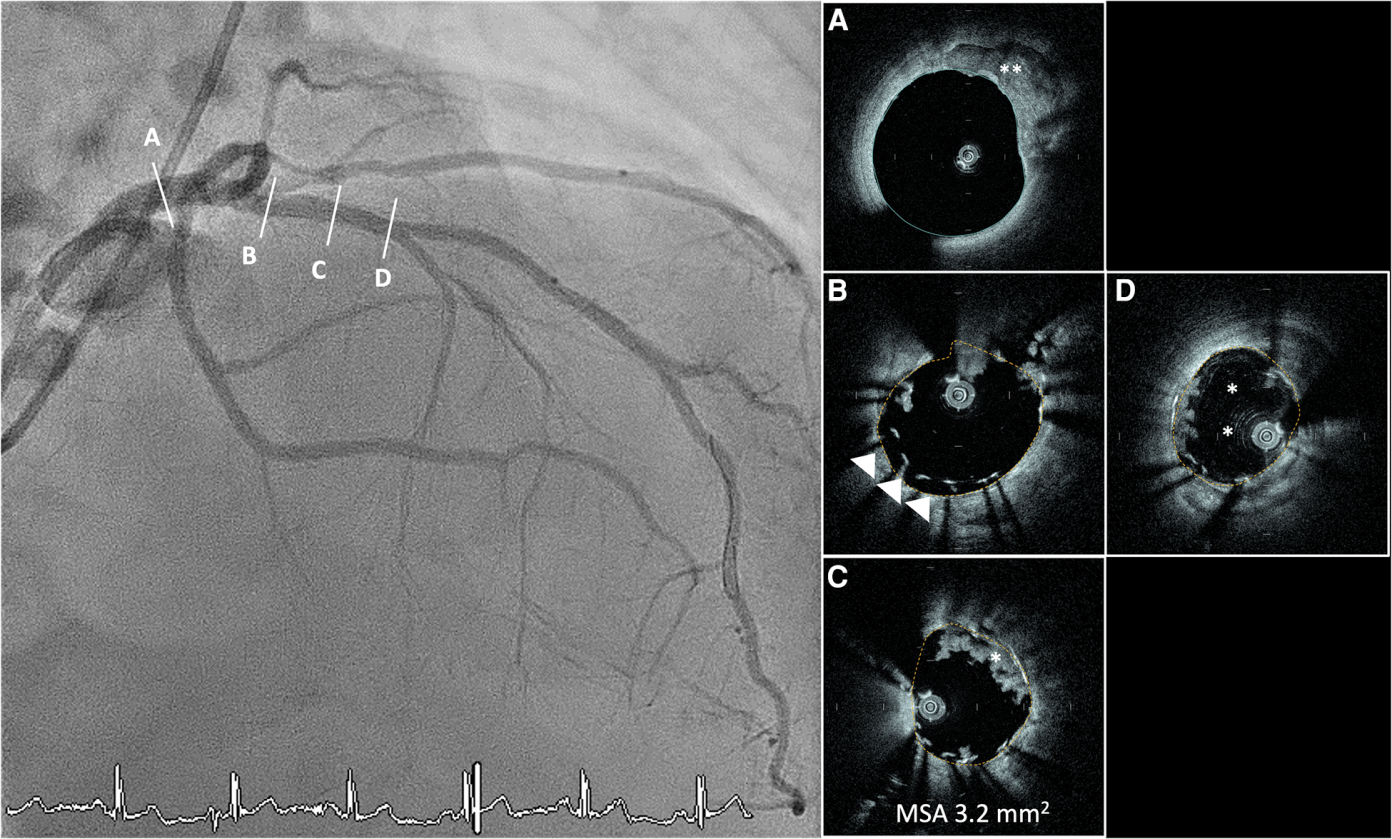
Hyperacute stent thrombosis in an unpreloaded patient with NSTEMI due to tight LAD stenosis. White arrow showing distal marker of the OCT catheter. (**A**) LM with calcification between 12 and 3 o’clock (**). (**B**) Proximal stent malapposition 6-9 o’clock. (**C**) Underexpanded stent (MSA 3.2 mm^2^) with intraluminal thrombus (*). (**D**) Distal stent edge with intraluminal thrombus 9-10 o’clock and circumferential calcium. NSTEMI, non-ST elevation myocardial infarction; LAD, left anterior descending artery; OCT, optical coherence tomography; LM, left main artery; MSA, minimum stent area.

**Figure 6:**
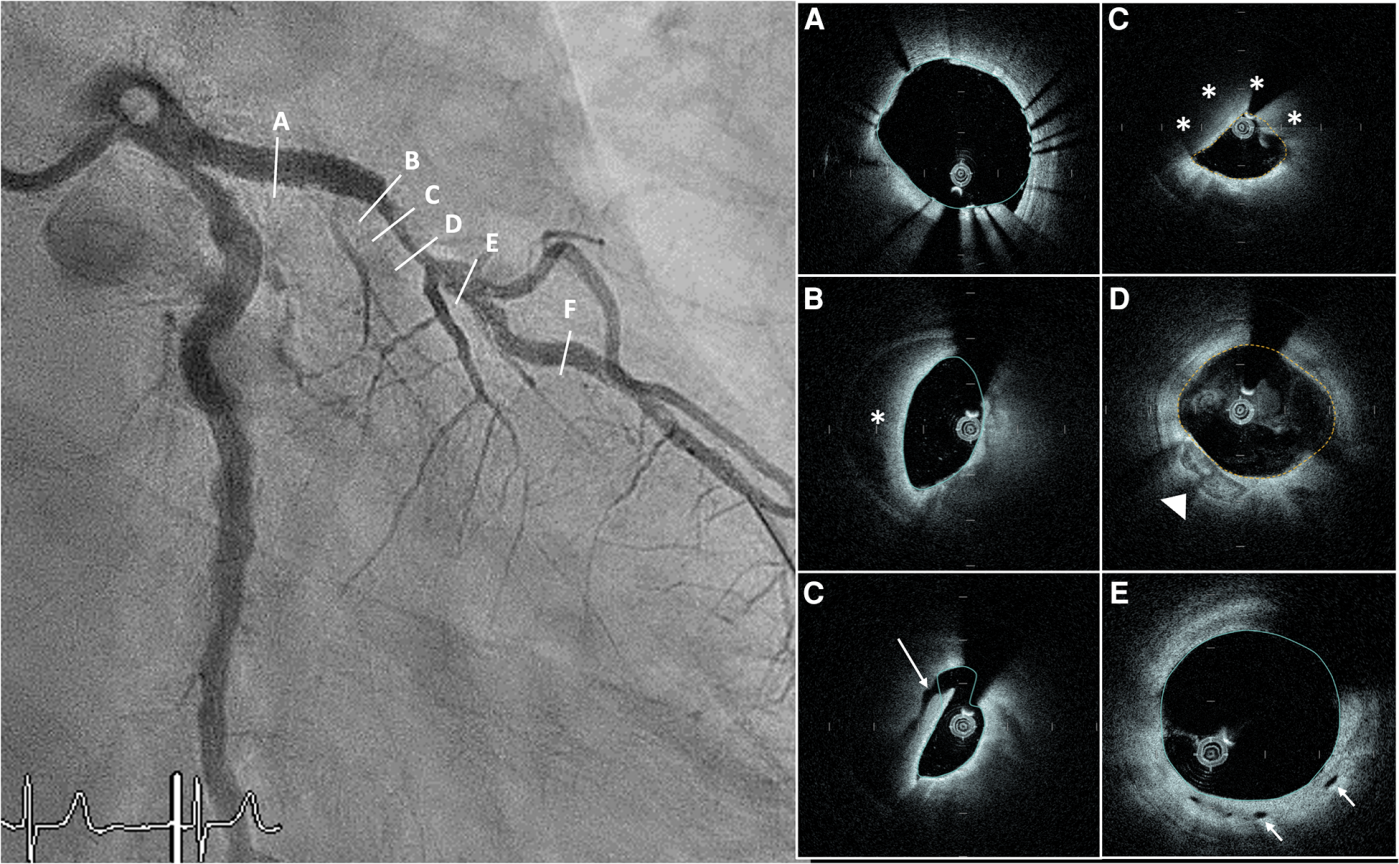
RAO-caudal view post angioplasty and stent implantation in the proximal LAD. Angiography depicting a tight hazy stenosis starting at the distal stent edge. (**A**) after stent implantation proximal with good stent apposition and expansion (MSA 10.5 mm2). (**B**) 180° crescent shaped hematoma just distal to the stent (*). (**C**) >180° dissection extending to the media (arrow). (**D**) 270°-degree hematoma (*) compressing the lumen (MLA 2.5 mm^2^). (**E**) Distal vessel showing no more hematoma and some calcified plaque at 7 o’clock (arrowhead). (**F**) Intimal vasculature (white arrow). RAO, right anterior oblique; LAD, left anterior descending artery; MSA, minimal stent area; MLA, minimal lumen area.

Overall, the catheter deliverability and crossability were excellent, and the image quality was very satisfactory. The Gentuity HF-OCT demonstrated similar imaging quality as compared to other OCT catheters for clear visualization of normal vascular anatomy and plaque morphologies (fibrotic, lipidic and calcific) (Figures 4, 5). The catheter was able to cross without predilatation a very tight lesion with a MLA of 0.4 mm^2^ (Figure 4 and Supplementary Video S1). In one patient with peri-interventional stent thrombosis in the context of ACS without P2Y_12_ preloading, HF-OCT allowed good visualization of intraluminal structures and defined potential future mechanical causes of stent failure such as stent under expansion and malapposition which were corrected (Figure 5). Figure 6 depicts a situation of stent edge hematoma and dissection which, according to the ILLUMIEN III criteria (2), was treated with additional stent implantation.

## 4 Discussion

In our case series, HF-OCT was able to image long coronary segments while maintaining good image quality, irrespective of vessel diameter, pre- vs. post-PCI condition, or lesion severity. However, image quality was influenced by tortuosity, with the absence of tortuosity giving better results than moderate or severe tortuosity. Even though 7 pullbacks did not meet the quality criteria of a CIL% ≥ 80%, operators were still able to analyze the images and use them for clinical decision-making. To our knowledge, the present analysis is the first study to examine the quality of HF-OCT images as a function of vessel tortuosity and diameter.

Our results are similar to those of another case series on pre-PCI HF-OCT images before any vessel preparation, which showed preserved image quality, even in serrated lesions, with a median CIL of 68.5 ± 14.9 mm and a CIL proportion of 87 ± 13%, as well as in post-PCI imaging (13). Our case series also confirm that image quality seems to persist in pre- vs. post-PCI conditions. However, the quality of our images was slightly lower. This could be because we had a different population with perhaps more complex lesions, or different operator experience. In both series, the global pullback quality of HF-OCT remained good with an overall CIL% ≥ 80%. Similarly, our results are in line with those of another study that compared HF-OCT with OCT, which had a global CIL for HF-OCT of 74 mm (71–75) (14).

Current OCT technologies are limited by larger diameter catheters (3-French), making navigation more difficult in very tight and/or tortuous and/or calcified lesions. Lesion preparation is often required prior to OCT, which may limit the value of pre-procedural diagnosis. In our case series, image quality was similar in all lesion severity categories, including lesions of larger-diameter vessels. As the HF-OCT catheter has a profile 2 times smaller than conventional OCT and can pass through very tight lesions that were previously unpassable without predilation, it is reassuring to note that the image remained satisfactory in these vessels. The use of this device may therefore enable better pre-PCI planning, by choosing a strategy based on plaque morphology prior to lesion preparation. Another advantage of the reduced profile would be the ability to perform complex PCI less invasively. Indeed, the Gentuity HF-OCT has recently been successfully used in a 4-French distal radial access, underlining its potential for minimally invasive treatments (17).

Our results may indicate that HF-OCT maintains consistent image quality in both wide and narrow sections of the same vessel, indicating its potential for imaging wider vessels (≥4 mm) without loss of quality. This raises the possibility of using HF-OCT for imaging wider vessels, such as the left main coronary artery (LMCA) or peripheral arteries. Although OCT offers better accuracy in plaque morphology and measurement of stent expansion than IVUS (6), the latter is more commonly used for imaging the LMCA (18), because of the better-studied nature of IVUS for LMCA, its deeper image penetration, and its reduced susceptibility to artefacts at the ostium as it does not require blood clearance (19). However, the extended scan range and faster pullback of HF-OCT, which reduce time needed of blood clearance (13, 14), could potentially offer an advantage in imaging the LMCA ostium, although this is purely speculative and requires evaluation in a dedicated study.

Our data suggests that increasing tortuosity is negatively associated with image quality, with a significantly lower CIL for moderate or severe tortuosity compared with no tortuosity. As the HF-OCT catheter was designed to navigate tortuous arteries difficult to reach with a conventional OCT catheter, this is, to our knowledge, the first study showing a negative association between tortuosity and image quality. While new technologies enable us to go even further in endovascular exploration, we believe that further research is needed on new imaging devices and on how vessel characteristics can influence image quality. This device seems to be suitable for practical use in intravascular imaging, while offering possible advantages over other available OCT catheters. However, the retrospective, single-arm nature of our study only allows us to generate hypotheses.

The HF-OCT system has however some limitations. While the high pullback speed is a definite advantage, it does come at the cost of image quality, and some operators noted difficulties in assessing fine plaque morphology such as thin-cap fibroatheroma plaques, macrophage, or cholesterol deposits. This first-generation system lacks an automated stent-detection system and algorithm for stent malapposition, and underexpansion, which is crucial to modern-day image-guided PCI. This will be improved with the second-generation software. The current system also lacks 3D reconstruction, limiting the assessment of complex vascular structures, stent failure, and in bifurcation stenting. The high pullback speed may limit the co-registration accuracy despite calibration. Moreover, the noise level may be distracting to some operators.

Our study is subject to several limitations that warrant careful consideration. The retrospective design, coupled with the limited sample size and absence of a control group, restricts ability to draw definitive conclusions and necessitates caution in interpreting the results. These constraints primarily allow for hypothesis generation rather than definitive assertions regarding the performance of the Gentuity® HF-OCT device. Moreover, the use of intravascular imaging was based on operator preference, which may introduce subjectivity and variability in both the application and interpretation of the imaging, as well as a risk of selection bias. Nonetheless, we believe our data confirm the validity of this promising technology will enhance its performance and likely broaden its use in the future.

## 5 Conclusion

The Gentuity® HF-OCT is a new device that can be used routinely for high-quality intravascular imaging, while offering distinct features and advantages over other available OCT catheters. It provides excellent visualization of vessel size, architecture, and plaque morphology for image-guided PCI treatments, even in stenotic or large vessels. Further software iterations will improve its performance and probably extend its use in the future.

## Data Availability

The raw data supporting the conclusions of this article will be made available by the authors, without undue reservation.
