# Supplementary material for: Experience with a novel high frequency optical coherence tomography device for intracoronary imaging: a case series
Source: Front Cardiovasc Med. 2024 Jun 7;11:1384222. doi: 10.3389/fcvm.2024.1384222 (PMC11190299; doi:10.3389/fcvm.2024.1384222)
Supplement: Supplementary file 1 [file Table1.docx]

**Supplementary material to:**

**Experience with a novel high frequency optical coherence tomography device for intracoronary imaging: a case series**

**By Garin D *et al.***

*Supplementary Table 1: indication of HF-OCT and main operator findings per patient.*

| Patient | Indication for HF-OCT | Main findings |
| --- | --- | --- |
| 1 | Pre-PCI diagnosis and HF-OCT-guided PCI on LAD | 2 tight fibro-fatty lesions on LAD |
| 2 | Pre-PCI diagnosis and HF-OCT-guided PCI on LCX | Proximal edge stent restenosis |
| 3 | HF-OCT-guided PCI on LAD | Good stent apposition |
| 4 | Pre-PCI diagnosis and HF-OCT-guided PCI on LAD | Acute distal stent dissection |
| 5 | Pre-PCI diagnosis | Good stent reendothelialization on RCA, LMCA and LAD |
| 6 | Pre-PCI diagnosis and HF-OCT-guided PCI on LAD | Multiple significant lesions on the LAD |
| 7 | HF-OCT-guided PCI on LAD | Good stent apposition |
| 8 | Pre-PCI diagnosis and HF-OCT-guided PCI on LAD | Very tight fibrotic lesion (passed without pre-dilatation) |
| 9 | HF-OCT-guided PCI on RCA and Pre-PCI diagnosis on LAD | Physiologically negative, anatomically stable LAD lesion |
| 10 | HF-OCT-guided PCI on LAD | Hyperacute stent thrombosis, treated with tirofiban infusion and stent optimization |
| 11 | Pre-PCI diagnosis | Good endothelialization of a stent in the ostial LAD protruding in LMCA |
| 12 | Pre-PCI diagnosis | Clinical and angiography suspected spasm but HF-OCT demonstrates an unstable tight stenosis in LAD. |
| 13 | Pre-PCI diagnosis and HF-OCT-guided PCI on LAD | Late stent failure with thrombosis due to stent underexpansion |
| 14 | Pre-PCI diagnosis and HF-OCT-guided PCI on LAD | Calcified lesion of the ostial LAD |

HF-OCT, high-frequency optical coherence tomography; PCI, percutaneous coronary intervention; LAD, left anterior descending artery; LCX, left circumflex artery; RCA, right coronary artery; LMCA, left main coronary artery.
